# Conventional versus drug-eluting beads chemoembolization for infiltrative hepatocellular carcinoma: a comparison of efficacy and safety

**DOI:** 10.1186/s12885-019-6386-6

**Published:** 2019-11-29

**Authors:** Zi-shu Zhang, Hui-zhou Li, Cong Ma, Yu-dong Xiao

**Affiliations:** Department of Radiology, the Second Xiangya Hospital of Central South University, No.139 Middle Renmin Road, Changsha, 410011 China

**Keywords:** Carcinoma, hepatocellular, Microspheres, Ethiodized oil, Chemoembolization, therapeutic

## Abstract

**Background:**

To compare the efficacy and safety between conventional transarterial chemoembolization (cTACE) and drug-eluting beads TACE (DEB-TACE) in patients with infiltrative hepatocellular carcinoma (iHCC).

**Methods:**

A total of 89 iHCC patients who were treated with either cTACE (*n* = 33) or DEB-TACE (*n* = 56) between April 2013 and September 2017 were included in this retrospective study. Patients with the situations that might have a poor outcome were defined as advanced disease including Child-Pugh class B, bilobar lesions, tumor size greater than 10 cm, ECOG 1–2, tumor burden of 50–70%, and the presence of ascites, arterioportal shunt (APS), and portal venous tumor thrombus (PVTT). The tumor response was measured 1-month and 3-month after the procedure. Progression-free survival (PFS) was calculated. Toxicity was graded by Common Terminology Criteria for Adverse Events v5.0 (CTCAE v5.0). The differences in tumor response, PFS, and toxicity were compared between the DEB-TACE group and cTACE group.

**Results:**

At 1-month and 3-month after the procedure, the objective response rate (ORR) in the overall study population was similar in DEB-TACE group and cTACE group. The disease control rate (DCR), at 1-month after the procedure, was significantly higher in the patients treated with DEB-TACE relative to those treated with cTACE (*P* = 0.034), while after 3 months, the difference did not differ between two groups. DEB-TACE showed a higher DCR than cTACE in patients with tumor size greater than 10 cm (*P* = 0.036) or associated with APS (*P* = 0.030) at 1-month after the procedure, while after 3 months, the difference was only noted in patients with APS (P = 0.036). The median PFS in DEB-TACE group was 96 days, while in cTACE group was 94 days, and there was no difference in PFS between two groups (*P* = 0.831). In the side effect analysis, abdominal pain (*P* = 0.034) and fever (*P* = 0.009) were more frequently present in the cTACE group than DEB-TACE group, but there was no difference in high grade liver toxicity between the two groups.

**Conclusions:**

Compared to cTACE, DEB-TACE offers slightly better DCR and tolerability for iHCC patients, particularly in patients associated with APS and large tumor size. However, DEB-TACE does not provide higher PFS than cTACE.

## Background

Hepatocellular carcinoma (HCC) is the sixth most common neoplasm and the third leading cause of cancer-related mortality worldwide, and it is an important medical problem as over 782,000 cases diagnosed and 746,000 deaths each year [1]. The morphology of HCC can be manifested as a nodular pattern, massive pattern, or diffuse/infiltrative pattern [2–4]. Infiltrative HCC (iHCC) represents 7–15% of HCC cases and is often associated with hepatitis B infection, particularly in Asian countries [5, 6]. The prognosis of iHCC is poor, and the therapeutic modality is limited [7–9]. Due to the high propensity of portal vein involvement, patients with iHCC are not candidates for curative treatments, such as transplantation, hepatectomy, and local ablation [10, 11]. Palliative treatment, such as transarterial chemoembolization (TACE), is occasionally shown to be beneficial for such patients [8, 9, 12, 13]. However, patients presenting with iHCC often have associated severe liver cirrhosis and liver function deterioration [14], thus, the reservation of liver function during TACE is counted as one of the first priority. Although it has been reported that TACE with drug eluting beads (DEB) offers higher intratumoral concentrations and lower systemic concentrations of doxorubicin compared to conventional TACE (cTACE), the efficacy of DEB-TACE relative to cTACE is rather controversial [15–17]. Therefore, the purpose of this study was to investigate and compare efficacy and safety between DEB-TACE and cTACE in patients with iHCC.

## Methods

### Study population

This single-institution retrospective study was approved by the Institutional Ethics Committee of the Second Xiangya Hospital of Central South University in accordance with Declaration of Helsinki (approved number: 2018S230). Verbal informed consent was obtained from patients or their family members.

**Fig. 1:**
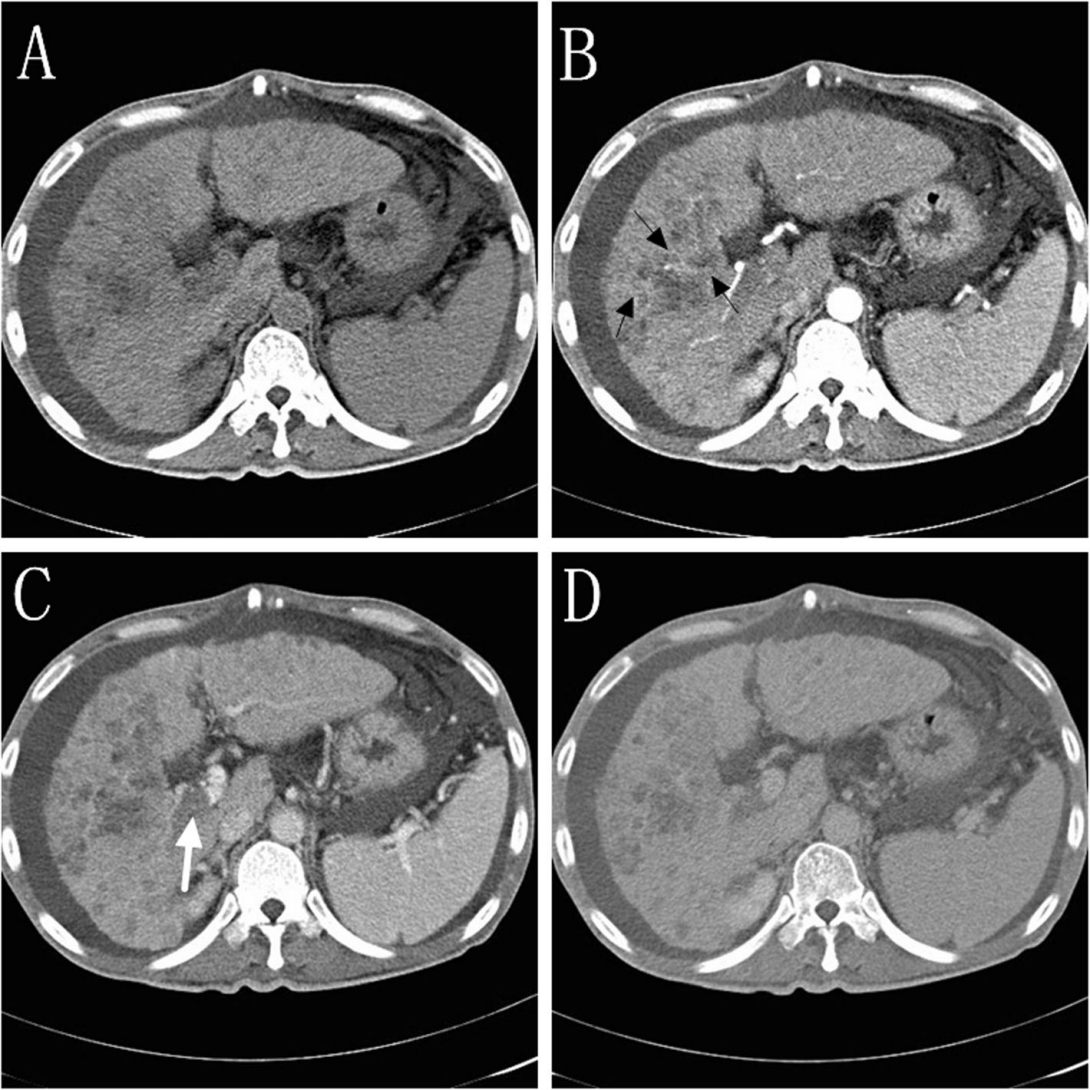
Images of a patient with infiltrative HCC (iHCC). Unenhanced CT image showing a low-density lesion in the right lobe of the liver (**a**), contrast-enhanced image showing the lesion slightly heterogeneously enhanced in the arterial phase with a “mosaic sign” (black arrow) (**b**); “washout” performance with tumor thrombus is noted in the right branch of the portal vein (white arrow) in the portal venous phase (**c**) and delayed phase (**d**)

**Fig. 2:**
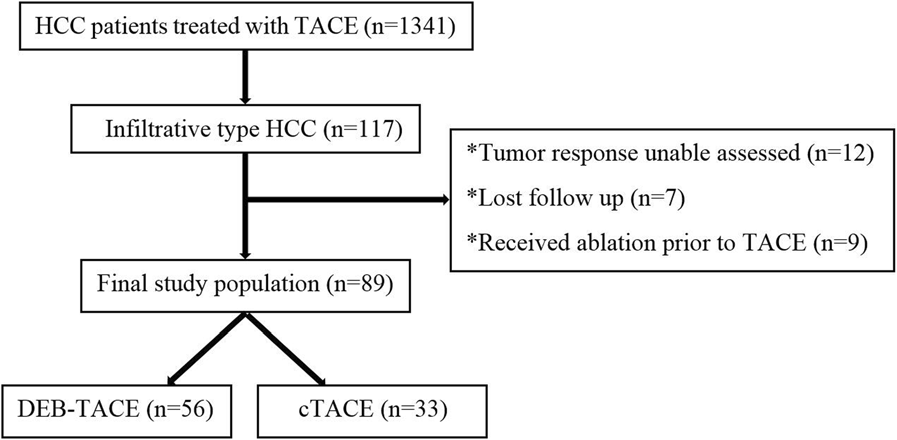
Diagram of the study population

A total of 1341 patients with HCC underwent TACE between April 2013 and September 2017 were included in this retrospective study. Of 1341 patients, 117 patients were iHCC. The definition of iHCC was presence of an extensive permeative hepatic tumor with ill-defined margins on computed tomography (CT) or magnetic resonance (MR) (Fig. 1) [10]. The exclusion criteria were as follows (Fig. 2): (a) patients in whom we were unable to assess the tumor response according to the modified Response Evaluation Criteria in Solid Tumors (mRECIST) for example the lesion was less than 1 cm, the lesion was unsuitable for repeat measurement, the lesion did not show intratumoral arterial enhancement on contrast-enhanced CT or MRI (*n* = 12); (b) patients who lost follow-up (*n* = 7); (c) patients received radiofrequency or microwave ablation prior to TACE (*n* = 9). Thus, the final study population consisted of 89 patients. Of these 89 patients, 56 patients were in the DEB-TACE group, and 33 patients were in the cTACE group. The data collected included patient age, patient gender, etiology of cirrhosis, Eastern Cooperative Oncology Group (ECOG) performance status, primary tumor size, Child-Pugh class, serum total bilirubin, creatinine and albumin levels, Barcelona Clinic Liver Cancer (BCLC) stage, model for end-stage liver disease (MELD) score, tumor burden, and presence or absence of portal venous tumor thrombus (PVTT), arterioportal shunt (APS), ascites, and distant metastasis. In the present study, patients with the situations that might have a poor outcome were defined as advanced disease including Child-Pugh class B, bilobar lesions, a tumor size greater than 10 cm, ECOG 1–2, a tumor burden of 50–70%, and the presence of ascites, arterioportal shunt (APS), and portal venous tumor thrombus (PVTT).

#### Interventions

The procedure was performed by three interventional radiologists with 17-year, 21-year and 13-year experience of liver interventions, respectively. The femoral artery was routinely catheterized. Celiac trunk arteriography and superior mesenteric arteriography, as well as indirect portography, were performed using a 4F catheter to demonstrate the variant hepatic arterial anatomy and evaluate the patency of the portal vein. Selective arteriography was performed to demonstrate the tumor blood supply, then a 2.7 F coaxial microcatheter (Progreat, Terumo Medical Corporation) was used to super-select the third- or fourth-order branch of the hepatic artery that supplied the target tumor. The chemoembolization was performed as selectively as possible. Lobar chemoembolization was performed only if the tumor feeding artery was difficult to super-select.

For the DEB-TACE group, the patients were treated with an intra-arterial injection of 1–2 g DEB (CalliSpheres Beads, Jiangsu Hengrui Medicine Co. Ltd). The size of DEB varied from 100 μm to 500 μm, and the amount of doxorubicin used ranged from 80 mg to 150 mg. The embolization endpoint was defined as stasis of blood flow in the tumor-feeding artery, which was often measured by the time it took to clear the contrast column (typically 4 heart beats). Repeated hepatic arteriography was performed to assess the devascularization after embolization. For the cTACE group, the patients were treated with an intra-arterial injection of 5–15 mL oil-doxorubicin emulsion. The oil-doxorubicin emulsion was created using the water-in-oil technique by mixing iodized oil (Lipiodol, Guerbet Group) with a distilled water solution containing a drug cocktail of dissolved doxorubicin at a ratio of 3:1. The doxorubicin dose used varied from 50 mg to 75 mg. After the oil-doxorubicin emulsion was injected, gelfoam slurry was used to embolize the tumor-feeding artery. The endpoint of embolization in the cTACE group was the same as that in the DEB-TACE group.

In both DEB-TACE and cTACE groups, follow-up imaging was performed 1–2 months intervals, repeat treatment was performed when there was viable tumor on the follow-up images. Patients received a maximum of three chemoembolizations (at baseline, 1 month, and 3 months) with a mean follow-up of 6 months.

#### Tumor response assessment

Tumor response was measured on contrast-enhanced CT or MR imaging based on the longest dimension of the viable tumor at 1 month and 3 months after the first TACE session according to mRECIST. Two liver radiologists with 18 years and 14 years of experience who were not involved in the treatment independently reviewed images and measured the longest dimension at before and 1 month as well as 3 months after the first TACE session. The diameter measured between two radiologists were averaged in each patient. The objective response rate (ORR) and disease control rate (DCR) of the two groups were calculated.

#### Progression-free survival (PFS) assessment

The PFS was defined as time between date of treatment and death whatever the cause, tumor progression or last clinical follow-up. The PFS was calculated and compared between DEB-TACE group and cTACE group.

#### Safety assessment

The safety assessment was evaluated based on a procedure related to toxicity grading. Toxicities were classified as clinical and laboratory toxicity, which was assessed within 1 month after the procedure. The grading of toxicity was defined as low (grades 1–2) or high (grades 3–4) according to Common Terminology Criteria for Adverse Events v5.0 (CTCAE v5.0).

#### Statistical analysis

Continuous data were expressed as the mean ± SD. All statistical analyses were conducted using a statistics package (SPSS version 20, International Business Machines Corporation), and *P* < 0.05 was considered statistically significant. Continuous data, such as age, primary tumor size, serum total bilirubin, creatinine, and albumin, and MELD score were compared between the two groups using a 2-tailed independent samples t-test. Categorical data, such as the ORR, DCR, etiology of cirrhosis, ECOG ps, Child-Pugh class, BCLC stage, tumor burden, and presence or absence of PVTT, ascites, distant metastasis, and APS, were compared between the two groups using the Chi-square test or Fisher’s exact test (if appropriate). PFS curve was performed using the Kaplan-Meier method and compared between two groups using the log-rank test. The intraclass correlation coefficient (ICC) was performed to evaluate the inter-reader agreement of diameter measurement between two radiologists. Agreement was classified as poor (ICC, 0–0.40), fair to good (ICC, 0.40–0.75), and excellent (ICC, > 0.75).

## Results

### Patient characteristics

The study population included 73 males and 16 females with a mean age of 55.6 ± 11.9 years and age range from 28 to 83 years. All patients presented with liver cirrhosis, and the etiologies of cirrhosis were hepatitis B virus infection (*n* = 76), hepatitis C virus infection (*n* = 8), alcohol abuse (*n* = 4), and primary biliary cirrhosis (*n* = 1). The diagnosis of iHCC was made based on pathology (biopsy, *n* = 21) or American Association for the Study of Liver (AASLD) practice guidelines (*n* = 68). In the entire study population, there were 30 patients with Child-Pugh class B (30/89, 33.7%), 47 patients with bilobar lesions (47/89, 52.8%), 46 patients with tumor size greater than 10 cm (46/89, 51.7%), 60 patients with ECOG 1–2 (60/89, 67.4%), 21 patients with tumor burden of 50–70% (21/89, 23.6%), and 37 patients with ascites (37/89, 41.6%), 63 patients with APS (63/89, 70.8%), and 74 patients with PVTT (74/89, 83.1%). Of the 89 patients, 84 patients were treatment-naive, whereas 5 patients received the treatment of combination of TACE and sorafenib. The clinical findings of the two groups are summarized in Table 1. There was no significant difference in the baseline data between the two groups.

**Table 1:** Clinical findings of DEB-TACE group and cTACE group

Variables	DEB-TACE (*n* = 56)	cTACE (*n* = 33)	*P* value
Age (years)	55.3 ± 11.6	56.1 ± 12.6	0.934
Gender (male/female)	45/11	28/5	0.594
Tumor size (cm)	11.2 ± 6.6	10.7 ± 5.8	0.588
Cirrhotic etiology (HBV/HCV/alcohol/PBC)	49/5/2/0	27/3/2/1	0.624
ECOG ps (0/1 + 2)	18/38	11/22	0.908
Total bilirubin (umol/L)	24.5 ± 25.3	17.2 ± 9.0	0.055
Albumin (g/L)	35.5 ± 5.0	35.7 ± 3.9	0.206
Creatinine (umol/L)	70.8 ± 23.9	74.3 ± 20.9	0.403
Bilobar lesions (yes/no)	32/24	15/18	0.286
Ascites (yes/no)	26/30	11/22	0.226
Tumor burden (<50%/50–70%)	41/15	27/6	0.356
Child-Pugh class (A/B)	36/20	23/10	0.602
MELD score	8.4 ± 1.7	8.8 ± 1.8	0.901
BCLC staging (B/C)	8/48	5/28	1.000
PVTT (yes/no)	47/9	27/6	0.797
Distant metastasis (yes/no)	9/47	7/26	0.542
APS (yes/no)	40/16	23/10	0.862
Received sorafenib (yes/no)	3/53	2/31	1.000

Note: *DEB-TACE* Drug-eluting beads transarterial chemoembolization, *cTACE* Conventional transarterial chemoembolization, *HBV* Hepatitis B virus, *HCV* Hepatitis C virus, *PBC* Primary biliary cirrhosis, *ECOG ps* Eastern Cooperative Oncology Group performance status, *Meld* Model for end-stage liver disease, *BCLC* Barcelona Clinic Liver Cancer, *PVTT* Portal venous tumor thrombus, *APS* Arterio-portal shunts

### Assessment of tumor response

There was excellent reproducibility of diameter measurement between two radiologists, with ICCs of 0.871 (before the first TACE), 0.813 (1 month after the first TACE), and 0.884 (3 months after the first TACE).

**Fig. 3:**
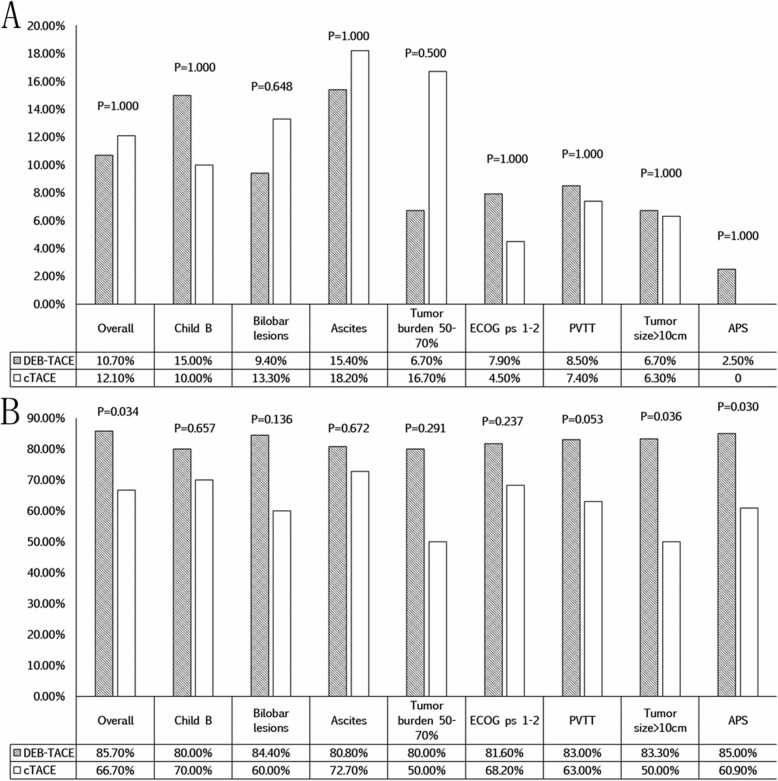
Tumor response at 1 month after the first TACE session. The objective response rate (ORR) (**a**) and disease control rate (DCR) (**b**) are shown. Note: CR, complete response; PR, partial response; SD, stable disease; PD, progressive disease; DEB-TACE, drug-eluting bead transarterial chemoembolization; cTACE, conventional transarterial chemoembolization; ECOG ps, Eastern Cooperative Oncology Group performance status; PVTT, portal venous tumor thrombus; APS, arterioportal shunt

At the first month, radiological evaluation of tumor response was available for all patients. In the DEB-TACE group, there were no patients with complete response (CR), 6 patients with partial response (PR), 42 patients with stable disease (SD), and 8 patients with progressive disease (PD). In the cTACE group, there were no patients with CR, 4 patients with PR, 18 patients with SD, and 11 patients with PD. The ORR (CR + PR) did not significantly differ between the DEB-TACE group and cTACE group (10.7% vs. 12.1%, *P* = 1.000). However, the DCR (CR + PR + SD) was significantly better in the DEB-TACE group than in the cTACE group (85.7% vs. 66.7%, *P* = 0.034). In the subgroups of advanced disease, there was no significant difference in the ORR between patients treated with DEB-TACE and those treated with cTACE (all *P* > 0.05). Nevertheless, for the patients with a tumor size greater than 10 cm (*P* = 0.036) and a presence of APS (*P* = 0.030), those in the DEB-TACE group showed a better DCR result than those in the cTACE group. The tumor response after the first month in the two groups is shown in Fig. 3.

**Fig. 4:**
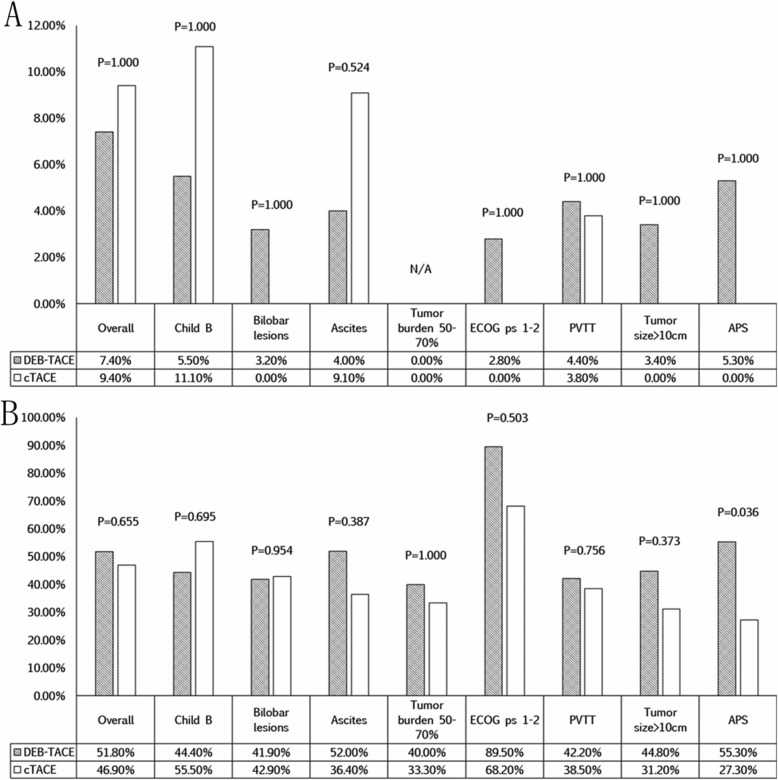
Tumor response at 3 months after the first TACE session. The ORR (**a**) and DCR (**b**) are shown

After 3 months, tumor response was available in only 86 patients because 3 patients died (DEB-TACE group, *n* = 2; cTACE group, *n* = 1). In the DEB-TACE group, there were no patients with CR, 4 patients with PR, 24 patients with SD, and 26 patients with PD. In the cTACE group, there were no patients with CR, 3 patients with PR, 12 patients with SD, and 17 patients with PD. There was no difference in both ORR and DCR between two groups. However, for patients with APS, those in the DEB-TACE group had a better DCR than those in the cTACE group (*P* = 0.036). The tumor response after 3 months in the two groups is shown in Fig. 4.

### Assessment of PFS

**Fig. 5:**
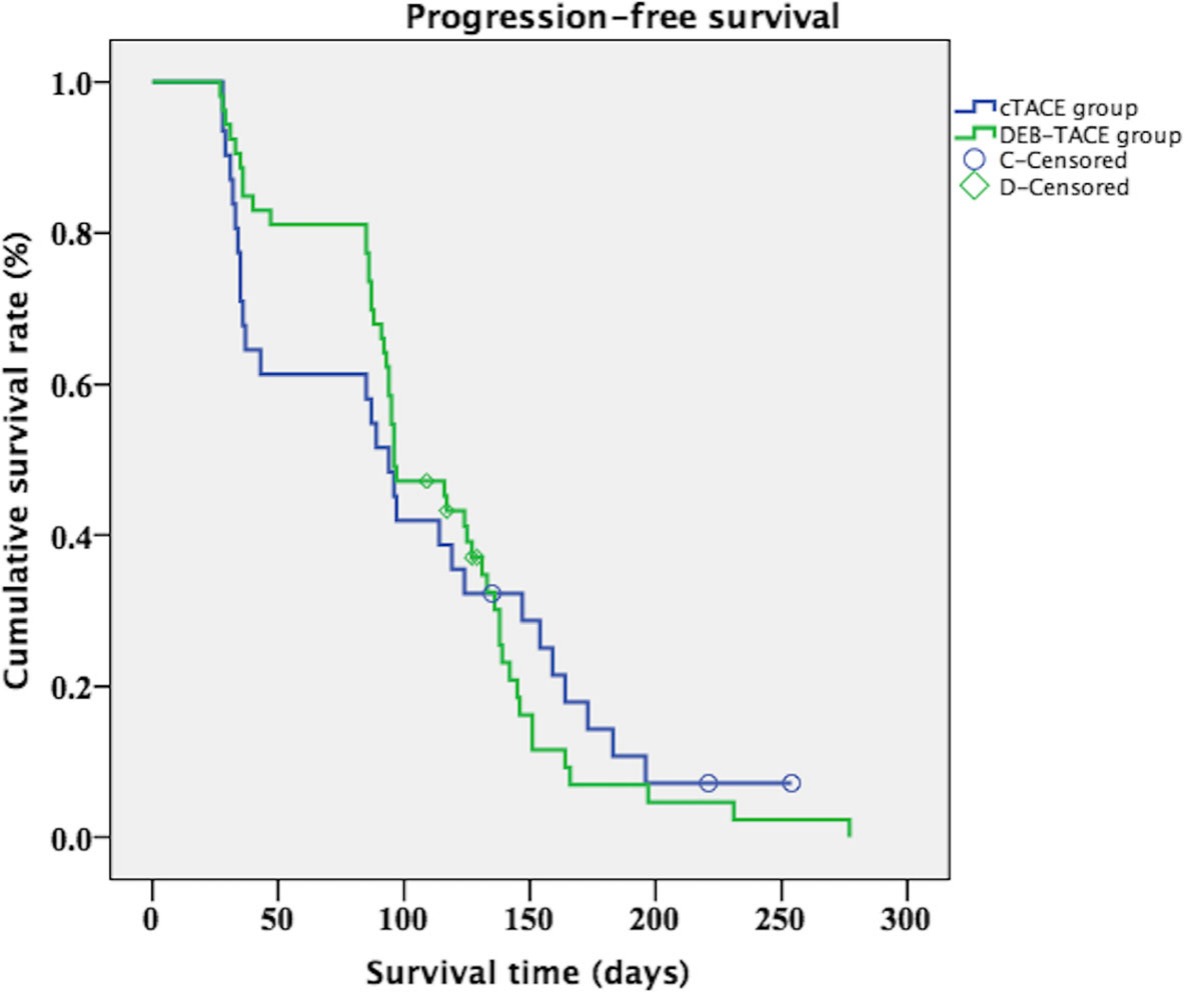
The progression-free survival of DEB-TACE group and cTACE group

Among the 89 patients, PFS was assessed in 84 patients because 5 patients received the combination therapy of sorafenib and TACE (DEB-TACE group, *n* = 3; cTACE group, *n* = 2). After a mean follow-up of 6 months, 77 patients (77/84, 91.7%) experienced tumor progression and 3 patients died. The median PFS in overall study population was 96 days, in DEB-TACE group was 96 days, while in cTACE group was 94 days. There was no difference in PFS between DEB-TACE group and cTACE group (*P* = 0.831). The PFS curve is shown in Fig. 5.

### Assessment of safety

The safety assessment was performed within 1 month after the procedure. The detailed safety assessment is shown in Table 2. The most common toxicities were moderate fever (*P* = 0.026) and abdominal pain (*P* = 0.034), which were more frequent in patients treated with cTACE than in those treated with DEB-TACE. Four patients (two in the DEB-TACE group and two in the cTACE group) developed hepatic failure, and two patients (one in the DEB-TACE group and one in the cTACE group) suffered mild gastrointestinal bleeding after the procedure. No deaths occurred within 1 month of treatment. However, three patients died due to hepatic failure (*n* = 2) or gastrointestinal bleeding (*n* = 1) within 3 months after the procedure.

**Table 2:** Toxicities of DEB-TACE group and cTACE group

Variables	DEB-TACE (*n* = 56)	cTACE (*n* = 33)	*P* value
Clinical toxicity (grade 1–2)			
Abdominal pain (yes/no)	38/18, 67.9%	29/4, 87.9%	0.034
Fever (yes/no)	22/34, 39.3%	21/12, 63.6%	0.026
Nausea (yes/no)	3/53, 5.4%	6/27, 18.2%	0.072
Diarrhea (yes/no)	3/53, 5.4%	4/29, 12.1%	0.416
Clinical toxicity (grade 3–4)			
Hepatic failure (yes/no)	2/54, 3.6%	2/31, 6.1%	0.625
Death (yes/no)	0	0	N/A
GI bleeding (yes/no)	1/55, 1.8%	1/32, 3.0%	1.000
Laboratory toxicity (grade 1–2)			
Elevated ALT (yes/no)	12/44, 21.4%	9/24, 27.3%	0.531
Elevated AST (yes/no)	5/51, 8.9%	8/25, 24.2%	0.064
Elevated ALP (yes/no)	10/46, 17.9%	6/27, 18.2%	0.969
Hyperbilirubinemia (yes/no)	17/39, 30.4%	9/24, 27.3%	0.757
Creatinine (yes/no)	0	0	N/A
Laboratory toxicity (grade 3–4)			
Elevated ALT (yes/no)	0	0	N/A
Elevated AST (yes/no)	0	0	N/A
Elevated ALP (yes/no)	0	0	N/A
Hyperbilirubinemia (yes/no)	1/55, 1.8%	3/30, 9.1%	0.142
Creatinine (yes/no)	0	0	N/A

Note: *DEB-TACE* Drug-eluting beads transarterial chemoembolization, *cTACE* conventional transarterial chemoembolization, *ALT* Alanine aminotransferase, *AST* Aspartate aminotransferase, *ALP* Alkaline phosphatase, *GI* Gastrointestinal

## Discussions

The performance of TACE for iHCC patients is rather controversial. Hung TH, et al. reported that TACE was a preferred therapy for iHCC patients [9], while Lopez RR Jr. et al. demonstrated no benefit of TACE for such patients [18]. In the present study, we evaluated the 1-month and 3-month tumor responses in patients with iHCC after undergoing DEB-TACE or cTACE, and the results showed that DEB-TACE might provide slightly better tumor response for patients with iHCC than cTACE. Regarding advanced disease, such as a tumor size greater than 10 cm (at 1-month, *P* = 0.036) and the presence of APS (at 1-month, *P* = 0.030; at 3-month, *P* = 0.036), DEB-TACE also showed an improved DCR compared to cTACE. Tumor size is a prognostic factor for HCC patients, and a previous study suggested that DEB-TACE is suitable for controlling large HCC lesions, while cTACE is suitable for small lesions [19–21]. In addition, in our clinical practice, we find that iHCC often shows a poor deposition of lipiodol during cTACE, and previous study also has similar findings [22]. This is presumably because of the presence of a hepatic APS in iHCC, which intra-arterial lipiodol will pass through into the portal vein, resulting in poor deposition in the tumor area [23]. Because DEB has the property of sustained tumor-selective drug delivery and permanent embolization of tumor-feeding vessels, we believe that DEB is more appropriate than lipiodol for patients with iHCC and APS [24–26].

An evaluation of toxicity showed that the common toxicities of two groups were low grade and self-limiting, which indicated that both DEB-TACE and cTACE were safe for iHCC patients. However, patients treated with DEB-TACE were less likely to have moderate fever and abdominal pain, which indicated that DEB-TACE was more tolerable for iHCC patients. These symptoms are those of postembolization syndrome, and previous studies have demonstrated a lower incidence secondary to DEB-TACE relative to cTACE [27–29]. Although patients treated with DEB-TACE received a relatively high dose of doxorubicin, there was no significant difference in both low-grade and high-grade laboratory toxicities between the two groups. This may be attributed to the higher concentrations of doxorubicin in the intratumoral area and lower concentrations in the systemic circulation when DEB-TACE is performed.

It should be noted that the evaluation of tumor response for iHCC is rather controversial [8, 12, 30, 31]. Although, as previous studies have described [30, 31], a radiological response of iHCC is difficult to obtain due to the poorly demarcated boundary of the tumor, in the present study, we found that iHCC with arterial phase enhancement on contrast-enhanced CT/MRI can be evaluated with mRECIST criteria. Besides, the excellent value of ICCs showed the high reproducibility of diameter measurement in iHCC, which was feasible in the determination of tumor response.

This study has several limitations. First, because of the short follow-up time in the present study, we have only assessed the PFS instead of overall survival of the two groups. Second, like all retrospective studies, the comparison of DEB-TACE and cTACE in patients with iHCC may be subject to selection bias. Besides, the drug dose in cTACE group was lower than those of DEB-TACE group. Further prospective studies with dose normalization should be conducted to confirm the efficacy and safety of these two patient population groups. Third, as 85.4% (76/89) of iHCC patients in the present study were classified as BCLC-C, only 6.6% (5/76) patients received sorafenib due to the high cost, even though sorafenib is the standard of care for patients with BCLC-C [1]. Last, in the present study, we only used *P* value to show the difference between two groups, no matched pair analysis was performed. Further study using matched pair analysis should be conducted to confirm the difference between cTACE group and DEB-TACE group in the management of iHCC.

## Conclusions

In conclusion, DEB-TACE may offer a slightly better tumor response than cTACE in patients with iHCC, particularly those associated with APS and a large tumor size. However the survival benefit of DEB-TACE in those patients is not confirmed by the present study.

## Data Availability

The datasets used and/or analyzed during the current study are available from the corresponding author on reasonable request.

## Abbreviations

AASLD: American Association for the Study of Liver
APS: Arterioportal shunt
BCLC: Barcelona Clinic Liver Cancer
CR: Complete response
CT: Computed tomography
cTACE: Conventional transarterial chemoembolization
CTCAE v5.0: Common Terminology Criteria for Adverse Events v5.0
DCR: Disease control rate
DEB: Drug eluting beads
ECOG: Eastern Cooperative Oncology Group
iHCC: Infiltrative hepatocellular carcinoma
MELD: Model for end-stage liver disease
MR: Magnetic resonance
mRECIST: modified Response Evaluation Criteria in Solid Tumors
ORR: Objective response rate
PD: Progressive disease
PFS: Progression-free survival
PR: Partial response
PVTT: Portal venous tumor thrombus
SD: Stable disease
TACE: Transarterial chemoembolization
